# Supplementary material for: Rapid turnover of life-cycle-related genes in the brown algae
Source: Genome Biol. 2019 Feb 14;20:35. doi: 10.1186/s13059-019-1630-6 (PMC6374913; doi:10.1186/s13059-019-1630-6)
Supplement: Supplementary file 3 — Protein alignments, corresponding Gblocks html file and CDS sequences for the lineage conserved gametophyte-biased genes. (ZIP 2109 kb) [file 13059_2019_1630_MOESM3_ESM.zip › Con_GA_alignments/Ec-05_004620.aa_ali.fasta-gb.htm]

Ec-05\_004620.aa.fasta.trans.aa\_ali.fasta


## Gblocks 0.91b Results

Processed file: **Ec-05\_004620.aa.fasta.trans.aa\_ali.fasta**  
Number of sequences: **2**  
Alignment assumed to be: **Protein**  
New number of positions: **270** (selected positions are underlined in blue)

```
                         10        20        30        40        50        60
                 =========+=========+=========+=========+=========+=========+
Ec-05_004620     MDGTRVFKTPPLLIAVPKQPMEEVFPAPLEGASKTLQLVSDGD--GNNNSQQHPHERSPM
MSTRG.52176.1    --GVRASKTPPLLIAVPRQ-SAEVLPAPLEGASKTLKQIGSVSYVDGDSSQRNPLERSLQ
                   #################   ##############             ###########


                         70        80        90       100       110       120
                 =========+=========+=========+=========+=========+=========+
Ec-05_004620     PVPAPFPPPQTITNGTSTSGSCLDGAGSRHDALLTDFSDRKSSERPSSLIGIGPDSPTFV
MSTRG.52176.1    PISAPSPPPQTIGSGICSS-KSYRGGGSAQEIVSTG--CRRSSERPSSLIGIGPESPTFI
                 ###################     ###########    #####################


                        130       140       150       160       170       180
                 =========+=========+=========+=========+=========+=========+
Ec-05_004620     SSPTGILTISTGLSPTKGPCKDSLASATSGSPGPVGYYPTSSYGQPSQIMAPMHAGAGSG
MSTRG.52176.1    SSPTGLLTISTGLSPTKDP-RESLASATSGSPGPVGYYPLSTIGQ-TLSMASTHAARG-D
                 ###################   #######################               


                        190       200       210       220       230       240
                 =========+=========+=========+=========+=========+=========+
Ec-05_004620     VFSPAAVFEPHNNIFGPSPPERPLYMTTRPAEVGSTLVEHRSQPDGTAPCTAAGLQQGEI
MSTRG.52176.1    I-----SSESHSNIFRPYPPENPLYMTIRPTEGAAAPREYGIHPIASTPDATTGLPQGQL
                         ####################################################


                        250       260       270       280       290       300
                 =========+=========+=========+=========+=========+=========+
Ec-05_004620     RTKDSLQGGQ---------------ATSLASLDSSSFFAMAPSTPLSSGSSSDLADLSQS
MSTRG.52176.1    KTEDVPGGGECYGYEEPGTKGTDSGGMSLASLDSSSFFAITPPPPPPS-TRGNHDDRCHP
                 #########                  #####################       #####


                        310       320       330       340       350       360
                 =========+=========+=========+=========+=========+=========+
Ec-05_004620     ELTSRMLRRSPTSATAAGGAKKLMVPLVAQQQRQATDNTDGYGALGGFDKTCNGRLVDGR
MSTRG.52176.1    ELERLLLCRSPD--TSGGGAQS------VELQHQATAANSGFAPK---IDTCGGRLIDGR
                 ###########                    ##########         ##########


                        370       380       390       400       410       420
                 =========+=========+=========+=========+=========+=========+
Ec-05_004620     GSSRFPAVVGYLGGGLAGGGSYGTIDGSSTQSGGGSLVVDAVDGAGRGCGESGNGSSEGE
MSTRG.52176.1    GCGSFP------------------------------------------------------
                 ######                                                      


                        430       440       450       460       470       480
                 =========+=========+=========+=========+=========+=========+
Ec-05_004620     GERHEDRIGSIAARVAPGKQPPRRLPSAVVLRTACNECAMKKTKCDGTKPCLSCRRKGVP
MSTRG.52176.1    ---------------------------------------------------LDVEKSHYS
                                                                    #########


                        490       500       510       520       530       540
                 =========+=========+=========+=========+=========+=========+
Ec-05_004620     CEFSLSQKKSKLPKPAKLPVPSTRNKTGRNMTAFREGGIISTPPSTVIGSLAGDGGTEGP
MSTRG.52176.1    CE----------------------------------------------------------
                 ##                                                          


                        550       560       570       580       590       600
                 =========+=========+=========+=========+=========+=========+
Ec-05_004620     SGRPGPPPGLSRPFAAYDGAPPNGGRGVRGSRGWRGARGGRGWRGGRVGRGGKGEYAVTG
MSTRG.52176.1    ------------------------------------------------------------
                                                                             


                        610       620       630       640       650       660
                 =========+=========+=========+=========+=========+=========+
Ec-05_004620     VGDGGDGAGPRGAVGIGDPTPSSSPGGAVSSRGGTSPPQGYGSARSSRSTPSSVFAPSHG
MSTRG.52176.1    ------------------------------------------------------------
                                                                             


                        670       680       690       700       710       720
                 =========+=========+=========+=========+=========+=========+
Ec-05_004620     VELASSSLAGVAPATSSPASSAPLSPRSGESTTPQSSRGAFSLRGPLRASSPAVRGASRG
MSTRG.52176.1    ------------------------------------------------------------
                                                                             


                        730       740       750       760       770       780
                 =========+=========+=========+=========+=========+=========+
Ec-05_004620     ISRGAAGGGLRGRGRGRGGHGKRRGRAGAGAVVSPLDSGSGPGFVEAESSLAGGNHLDRD
MSTRG.52176.1    ------------------------------------------------------------
                                                                             


                        790       800       810       820       830       840
                 =========+=========+=========+=========+=========+=========+
Ec-05_004620     DYEADGGSLGQKGASENCITTFAGGKQGQEQRDPTSRQLQGSPPQAQPGQRQAVPAASGL
MSTRG.52176.1    ------------------------------------------------------------
                                                                             


                        850       860       870       880       890       900
                 =========+=========+=========+=========+=========+=========+
Ec-05_004620     PDEKGEEVINRGDGRNEILFPTMYRHQQQPIPHGGGQQQHRVNANQSKATLVGKVEEKNR
MSTRG.52176.1    ------------------------------------------------------------
                                                                             


                        910       920       930       940       950       960
                 =========+=========+=========+=========+=========+=========+
Ec-05_004620     DEGRTEDETAKGAPTESDLLQHKIRDGTLFGIGHFGGIGGLDGYSLEASPAAATSATAGE
MSTRG.52176.1    ------------------------------------------------------------
                                                                             


                        970       980       990      1000      1010      1020
                 =========+=========+=========+=========+=========+=========+
Ec-05_004620     EESNDRRHATGYSGSYQARREQGSDDDGGDARDRNGRVGGDEDGIKHSLGAGGSSGGNSR
MSTRG.52176.1    ------------------------------------------------------------
                                                                             


                       1030      1040      1050      1060      1070      1080
                 =========+=========+=========+=========+=========+=========+
Ec-05_004620     SSGGGSSGSSSEASSDGDISGGEGDGREDGPRREMARGGGDRGDNMEAEGWGSQDEEEGN
MSTRG.52176.1    ------------------------------------------------------------
                                                                             


                       1090      1100      1110      1120      1130      1140
                 =========+=========+=========+=========+=========+=========+
Ec-05_004620     DGEDGNDEDEEDDEDEFAAQGEASGLGGGRIINKSTTVGIIISKSRPRRPSRKKTKKPVW
MSTRG.52176.1    ------------------------------LVRQSS------------------------
                                                                             


                       1150      1160      1170      1180      1190      1200
                 =========+=========+=========+=========+=========+=========+
Ec-05_004620     SQLRKACDRCTSKKVRCDGKEGCFQCSRVGATCHFSICRRSGPRDSNGHKTGTIGPIIPR
MSTRG.52176.1    ------------------------------------------------------------
                                                                             


                       1210      1220      1230      1240      1250      1260
                 =========+=========+=========+=========+=========+=========+
Ec-05_004620     DGYEKVTGRRGSCRISSQCRFVSADGQSKTEHYHCRAPGCEYASKTMEELMAHRKNHPEQ
MSTRG.52176.1    ------------------------------------------------------------
                                                                             


                       1270      1280      1290      1300      1310      1320
                 =========+=========+=========+=========+=========+=========+
Ec-05_004620     TPARLHRPVVATAIKQQAQKPKRILPQQLADSQGTLPPQQRQKKQRTAPPPPPPIPQPMP
MSTRG.52176.1    ------------------------------------------------------------
                                                                             


                       1330      1340      1350
                 =========+=========+=========+==
Ec-05_004620     PAASRPRPQSRQGTAPPPALLVVQRYSDFGNG
MSTRG.52176.1    -------------------------SLDILLD
```

```
Parameters used
Minimum Number Of Sequences For A Conserved Position: 2
Minimum Number Of Sequences For A Flanking Position: 2
Maximum Number Of Contiguous Nonconserved Positions: 8
Minimum Length Of A Block: 10
Allowed Gap Positions: None
Use Similarity Matrices: Yes
```

```
Flank positions of the 12 selected block(s)
Flanks: [3  19]  [23  36]  [50  79]  [85  95]  [100  139]  [143  165]  [189  249]  [268  288]  [296  311]  [332  341]  [351  366]  [472  482]  

New number of positions in Ec-05_004620.aa.fasta.trans.aa_ali.fasta-gb:  270  (19% of the original 1352 positions)
```
